# Clinical Study on Postoperative Treatment for Patients at High Risk of Oral Squamous Cell Carcinoma Recurrence

**DOI:** 10.7759/cureus.78395

**Published:** 2025-02-02

**Authors:** Kei-ichiro Miura, Mitsunobu Otsuru, Hiromasa Fukushima, Keisuke Omori, Tomofumi Naruse, Masahiro Umeda, Tomohiro Yamada

**Affiliations:** 1 Department of Oral and Maxillofacial Surgery, Nagasaki University Graduate School of Biomedical Sciences, Nagasaki, JPN; 2 Department of Oral and Maxillofacial Surgery, Kanagawa Dental University, Yokosuka, JPN

**Keywords:** concurrent chemoradiotherapy, efficacy, oral squamous cell carcinoma, postoperative treatment, recurrence

## Abstract

Background: Surgery is the standard treatment for oral squamous cell carcinoma (OSCC), and concurrent chemoradiotherapy (CRT) is recommended in cases where extranodal extension (ENE) or positive margins are found histopathologically after surgery. However, the indications and efficacy of CRT remain controversial. In this study, we investigated the efficacy of postoperative treatment by examining risk factors for postoperative OSCC recurrence.

Methodology: We investigated the postoperative treatment and prognosis of 52 patients with OSCC with high-risk factors for recurrence (28 with ENE only, 17 with positive margins only, and seven with both). ENE was classified into minor ENE (ENEmi < 2 mm) and major ENE (ENEma ≥ 2 mm).

Results: The prognosis for ENEmi was good regardless of whether postoperative treatment was administered; however, the prognosis for ENEma was significantly poor. In the ENEma group, the prognosis of the patients who underwent radiotherapy (RT) or CRT was better than that of patients who did not undergo postoperative treatment, with no significant differences between the RT and CRT groups. In patients with positive margins, the prognosis was better in those who underwent additional resection than in those who underwent CRT.

Conclusions: Patients with ENEma have a poor prognosis and require additional treatment with RT or CRT. Re-resection may improve the prognosis in patients with positive margins.

## Introduction

Oral squamous cell carcinoma (OSCC) is the 16th most common cancer and a relatively rare malignant neoplasm [1]. Although its incidence is high in some countries in South and Southeast Asia, it is rare in Japan, accounting for only 1%-2% of all cancers. According to the National Comprehensive Cancer Network (NCCN) guidelines, surgery is recommended as the primary treatment for OSCC, with concurrent chemoradiotherapy (CRT) or radiotherapy (RT) advised if extranodal extension (ENE) is present on postoperative histopathological examination; re-resection is recommended if positive margins are found, with consideration for RT or CRT if negative margins are achieved, if necessary [2].

ENE is a poor prognostic factor for OSCC [3,4]. Recent studies have classified ENE into two types: major ENE (ENEma), in which the infiltration extends >2 mm beyond the capsule, and minor ENE (ENEmi), in which the extension is ≤2 mm. In the report by Joshi et al., it was shown that ENEma had a worse prognosis than ENEmi in terms of overall survival and disease-free survival [5]. Similarly, Higaki et al. reported the same findings, and it has recently become clear that this classification is clinically very important [6]. On the other hand, how prognosis changes with treatment within each classification remains unclear. Yamada et al. also classified the degree of ENE into three types based on the tissue at the infiltrative edge and reported a poor prognosis in type C cases with extension into the perinodal fat or muscle tissue [7]. However, reports have also suggested no difference in prognosis based on the degree of ENE, making the significance of ENE classification controversial [8-10]. While some studies recommend additional resection for cases with positive margins [11], the rationale for RT remains unclear [12,13]. This study retrospectively reviewed patients with OSCC and reevaluated postoperative adjuvant treatment for high-risk cases of recurrence.

## Materials and methods

Study design and patients

Patients included in this study were 18 years or older at the time of OSCC diagnosis and had visited the Department of Oral and Maxillofacial Surgery at Nagasaki University Hospital between January 1, 2011, and December 31, 2021. They were diagnosed with OSCC, underwent surgical treatment, and had either ENE-positive lymph nodes or positive surgical margins. Only primary OSCC cases were included, and patients with a history of chemotherapy, radiotherapy, molecular-targeted therapy, or immune checkpoint inhibitors for the primary site, neck, or any other cancer were excluded. Additionally, patients who had received neoadjuvant therapy for OSCC or had recurrent cervical lymph node metastases were also excluded from the study. In addition, during surgery, we evaluated the margins using frozen section samples. Generally, if a positive margin was detected, additional resection was performed until a negative margin was achieved, except in anatomically high-risk areas.

Factors examined

The following information was obtained from medical records: age at first diagnosis, sex, primary site, clinical TN stage (UICC 8th edition), resection margin status (positive or negative), histopathological differentiation (well, moderate, or poor), number of metastatic lymph nodes, presence or absence of ENE with subclassification (ENEmi or ENEma), post-treatment details, and prognosis.

Pathological criteria

**Figure 1:**
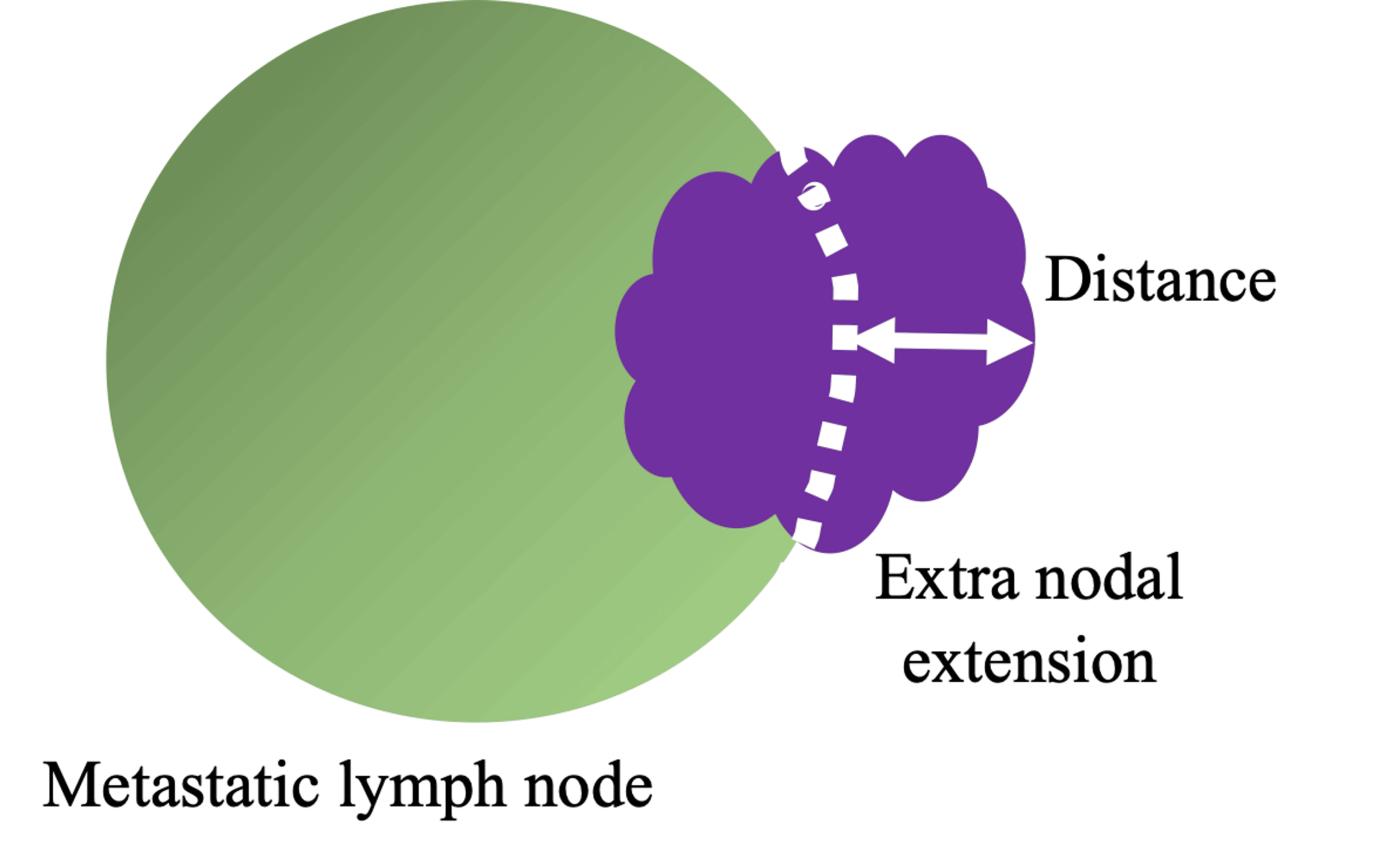
Extranodal extension. Green area: Metastatic lymph node. Purple area: Extranodal extension site. Dotted line: Hypothetical capsule surface. Double-headed arrow: Distance between the hypothetical capsule surface and the leading edge of extranodal invasive tumor cells. ENEmi was defined as tumor cell infiltration of less than 2 mm from the hypothetical capsule surface, while ENEma was defined as 2 mm or more. Image credit: Hiromasa Fukushima.

When tumor cells were present at the surgical margin, it was defined as a positive surgical margin, whereas when tumor cells were more than 5 mm away from the surgical margin, it was defined as a negative surgical margin. ENE was evaluated according to the AJCC guidelines. ENEmi was defined as tumor cell infiltration of <2 mm from the capsule, while ENEma was defined as infiltration of 2 mm or more (Figure 1). 

Criteria for postoperative treatment

Based on the NCCN guidelines, CCRT with high-dose cisplatin (CDDP) was administered postoperatively in cases where pathological examination confirmed ENE of metastatic cervical lymph nodes, with or without positive surgical margins. The postoperative treatment regimen consisted of three-weekly CDDP combined with RT. CDDP at a dose of 100 mg/m² was administered on day 1, day 22, and day 43. RT was delivered using a fractionated method, with a dose of 2 Gy per session, administered once per day, five days a week, for a total cumulative dose of 66 Gy. For cases with positive surgical margins only, additional resection was considered whenever feasible. If the margins were confirmed to be negative after additional resection, RT was considered. However, if additional resection was not possible, CCRT was considered. In patients with multiple metastases, metastases to levels IV-V, perineural invasion, or vascular invasion, and moderate risk factors for recurrence, RT was generally performed as a standard approach. Even in cases where CCRT was indicated, RT alone was performed in patients over 75 years of age or those with poor general health conditions, based on a comprehensive evaluation.

Statistical analysis

SPSS version 26.0 (Japan IBM Co., Ltd., Tokyo, Japan) was used for statistical analyses. The prognosis was analyzed using the Kaplan-Meier method and log-rank tests for overall survival (OS), disease-specific survival (DSS), locoregional control (LRC), and disease-free survival (DFS). A *P*-value < 0.05 was considered statistically significant.

Ethics and registration

This study adhered to the ethical guidelines of the Declaration of Helsinki and the Ethical Guidelines for Medical and Health Research Involving Human Subjects, as established by the Ministry of Health, Labour and Welfare of Japan. Ethical approval was obtained from the Institutional Review Board (IRB) of Nagasaki University Hospital (#24021905). As this was a retrospective study, patient-identifiable information was removed, and the research plan was published on the homepages of the Nagasaki University Hospital website with an opt-out option according to IRB instructions. This study was registered with the University Hospital Medical Information Network; the registration number was UMIN000049956, and the date of publication was January 5, 2023.

## Results

Patient characteristics

Of the 381 primary cases treated at our department during the study period, 52 cases were enrolled in the study. Patient characteristics are shown in Table 1.

**Table 1:** Patient characteristics. ENE, extranodal extension; ENEmi, ENE minor; ENEma, ENE major; CRT, chemoradiotherapy; RT, radiation therapy

Variable	Number of patients
Age (years)	26-94 (Median 66)
Sex	
Male	33
Female	19
Site	
Tongue	22
Maxilla and hard palate	9
Mandible	10
Oral floor	3
Buccal mucosa	5
Lower lip	1
Primary intraosseous carcinoma	2
T stage	
T1	3
T2	8
T3	13
T4	26
Primary intraosseous carcinoma	2
N stage	
N0	13
N1	6
N2	30
N3	3
Margin status	
Negative	28
Positive	24
Differentiation	
Well	43
Moderate	7
Poor	2
Degree of ENE	
ENEmi	7
ENEma	28
Number of positive nodes	
0	11
1-3	25
≧4	16
High-risk factor for recurrence	
ENE only	35
Positive margin only	24
Both ENE and positive margin	7
Postoperative treatment for ENE only	
CRT	12
RT	7
No treatment	8
Postoperative treatment for positive margin only	
Re-resection	12
CRT	5
Postoperative treatment of both ENE and positive margin	
Re-resection + CRT	1
CRT	3
No treatment	3

There were 33 males and 19 females, with a male-to-female ratio of 1.7:1. Patient ages ranged from 26 to 94 years, with a median age of 66 years. The most common primary tumor site was the tongue (22 patients), followed by the lower jaw gingiva, maxillary gingiva, and hard palate. There were several advanced cancers, including 11 cases of T1-2 and 39 cases of T3-4. ENE was observed in 35 patients, of which seven were ENEmi and 28 were ENEma. Positive margins were observed in 24 cases. High-risk factors for recurrence seen in the cases were ENE (*n* = 28), positive margins (*n* = 17), and both (*n* = 7).

Postoperative treatment and prognosis

**Figure 2:**
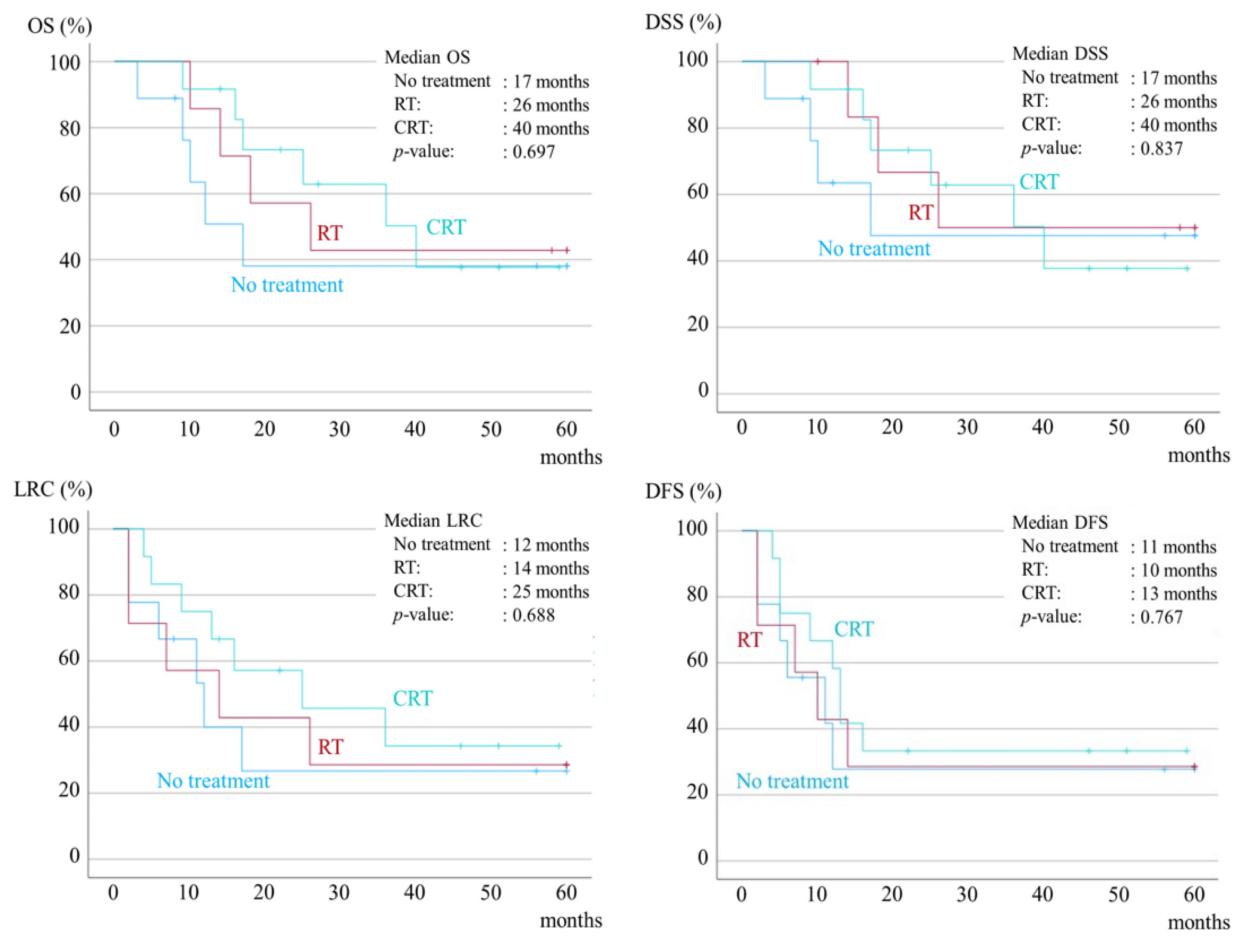
Kaplan-Meier survival curves for five-year overall survival (OS), five-year disease-specific survival (DSS), five-year loco-regional control (LRC), and five-year disease-free survival (DFS) according to postoperative treatment for ENE-only patients. RT, radiation; CRT, chemoradiotherapy; ENE, extranodal extension

Postoperative treatment for ENE-only patients included CRT in 12 patients, RT in seven, and no treatment in nine. There was a trend toward an increased survival time in patients who underwent CRT compared to those who received no treatment, but there were no significant differences in OS, DSS, LRC, or DFS (Figure 2).

**Figure 3:**
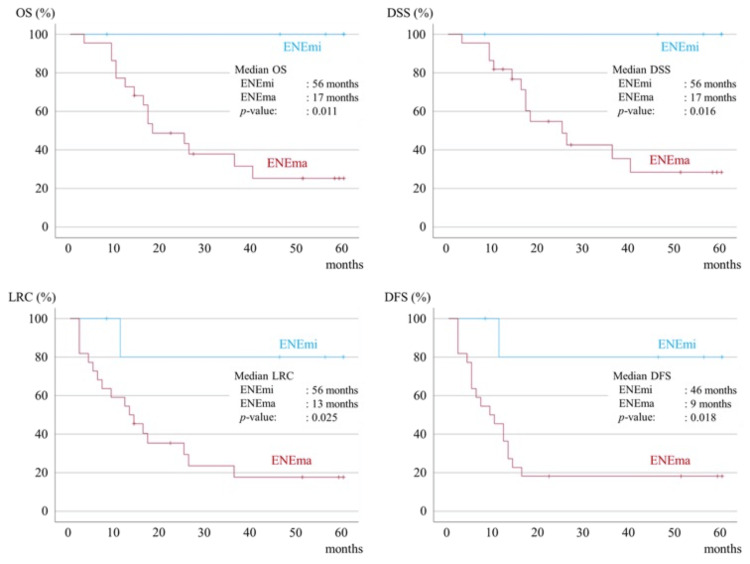
Kaplan-Meier survival curves for five-year OS, five-year DSS, five-year LRC, and five-year DFS according to the level of progression of ENE. OS, overall survival; DSS, disease-specific survival; LRC, loco-regional control; DFS, disease-free survival; ENE, extranodal extension; ENEmi, ENE minor; ENEma, ENE major

When the prognoses of the 28 ENE-only patients were calculated separately for ENEmi and ENEma, the prognosis for ENEma was significantly poorer in terms of OS, DSS, LRC, and DFS (Figure 3).

**Figure 4:**
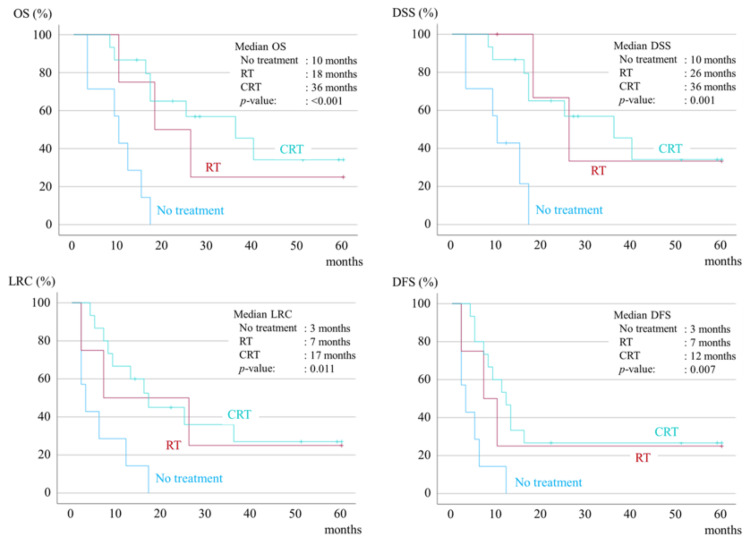
Kaplan-Meier survival curves for five-year OS, five-year DSS, five-year LRC, and five-year DFS in the ENEma group. OS, overall survival; DSS, disease-specific survival; LRC, loco-regional control; DFS, disease-free survival; ENE, extranodal extension; ENEma, ENE major; RT, radiation; CRT, chemoradiotherapy

The prognosis of ENEmi cases was good, and there was no difference in prognosis between the different treatment methods. In contrast, the prognosis for CRT or RT was significantly better in the ENEma group than in the no-treatment group (Figure 4).

**Figure 5:**
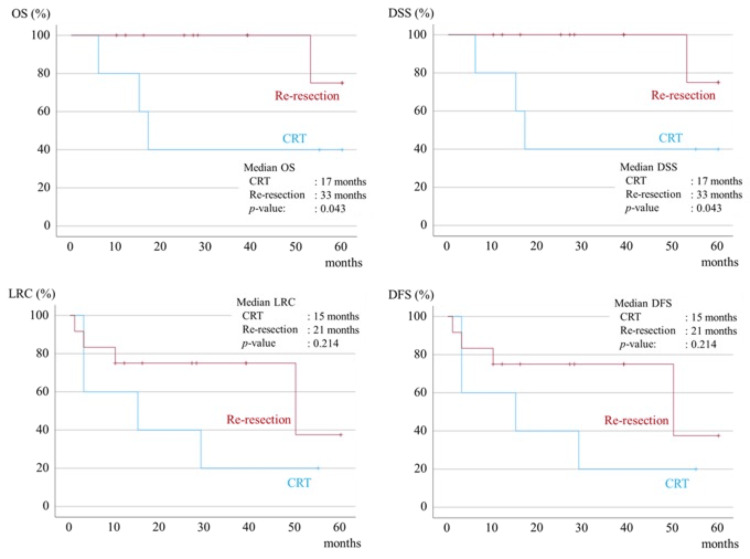
Kaplan-Meier survival curves for five-year OS, five-year DSS, five-year LRC, and five-year DFS in the positive margins group. OS, overall survival; DSS, disease-specific survival; LRC, loco-regional control; DFS, disease-free survival; CRT, chemoradiotherapy

Treatment for positive margins included re-resection in 12 patients and CRT in five, with the prognosis in the additional resection group being better than that in the CRT group. A significant difference was observed between the OS and DSS groups (Figure 5).

For patients with both ENE and positive margins, one underwent CRT after re-resection, three underwent CRT, and three did not receive any treatment. However, the small number of cases meant that it was not possible to compare the prognosis for each treatment method. In addition, owing to the small number of cases, multivariate analysis could not be performed.

## Discussion

The standard treatment for squamous cell carcinoma of the head and neck is surgical resection. If a positive margin or ENE is found histopathologically after surgery, RT is recommended with high-dose CDDP, based on the results of two large-scale RCTs conducted in 2004 [14,15]. However, these results were derived from clinical studies targeting head and neck cancer as well as oral cancer. Cooper subsequently examined the long-term prognosis of this treatment method and reported that while locoregional control improved in cases with positive margins or ENE, there was no improvement in OS [16]. In a phase 2 study, Kiyota et al. [17] reported that concurrent CRT for high-risk patients after surgery had a low effect on oral cancers. Additionally, in a study that only included patients with oral cancer, Yanamoto et al. [18] examined 118 cases with ENE or positive margins and reported that compared to RT alone, locoregional control improved with CRT, but there was no improvement in OS or DFS. The effectiveness of postoperative CRT for oral cancer is controversial; one reason for this is thought to be the lack of detailed studies that have examined the high-risk factors for recurrence. In the current study, we divided the high-risk factors for recurrence into ENE and positive margins and further divided ENE into ENEmi and ENEma, followed by an examination of the effectiveness of treatment methods based on these factors.

The prognosis for ENEmi was good, and there was no difference in OS or DSS between the CRT group and the group of patients who did not receive CRT. In contrast, patients with ENEma showed significantly better outcomes when they underwent RT or CRT than when they did not receive any further treatment. However, there was no difference in the prognosis between RT alone and CRT, and the effect of adding cisplatin was unclear, likely because of the small number of cases. Some factors to consider include the fact that ENEma refers to a condition where the tumor has clearly infiltrated beyond the lymph node (>2 mm), indicating a high biological malignancy and the possible presence of subclinical metastases. Furthermore, among patients for whom CRT was indicated but RT was chosen instead, it is likely that many were elderly or had poor overall health conditions. Previous studies have reported that CRT improved disease-free survival in patients with ENEma [19]. In the future, we need to increase the number of cases and conduct a detailed examination of the effects of each treatment.

The prognosis of patients with positive margins who underwent re-resection was better than that of patients who underwent CRT. The reasons for this can be considered as follows. First, additional resection ensures complete removal of the tumor, reducing the risk of residual tumor, whereas CRT carries a risk of tumor cell persistence, particularly the survival of radiation-resistant tumor cells. Second, additional resection allows for a more precise assessment of tumor extent and depth. When positive surgical margins are present, certain infiltration patterns such as perineural invasion or lymphovascular invasion may be overlooked. However, with additional resection, an appropriate evaluation of the resection area can be performed, thereby improving local control accuracy. Furthermore, CRT alone does not provide histological confirmation of tumor control, making it difficult to assess whether the tumor has been completely eradicated. Several factors influence the success of re-resection. Sufficient normal tissue must remain for additional resection to be feasible. Moreover, if the time elapsed since the initial surgery is short, scarring is minimal, making re-resection easier. However, as time passes, fibrosis progresses, making it difficult to distinguish between tumor and scar tissue, which may reduce the success rate of additional resection. Additionally, functional and aesthetic defects resulting from additional resection may significantly impair swallowing, phonation, and mastication functions. If reconstructive surgery is feasible, re-resection can be performed, but if reconstruction is not possible, CRT is prioritized. Also, the patient’s overall health condition is crucial in determining whether they can withstand the invasiveness of reoperation. As these are the results of the univariate analysis, there is a possibility of bias in the selection of treatment methods; because of this, it is not possible to draw any definitive conclusions. There is a need for further investigation into the selection criteria for the post-treatment of oral cancer.

This study had some limitations. As this was a single-center study with a small number of cases, it is unclear whether the results obtained can be generalized. Additionally, because this was a retrospective rather than an interventional study, there is the possibility of bias in the selection of each treatment method. Furthermore, in this study, the imbalance in gender distribution, the bias in primary tumor sites, and the possibility that age groups may have influenced the treatment cannot be completely ruled out. Also, the opt-out method may introduce selection bias by disproportionately including certain groups or, conversely, excluding others. However, as it is difficult to randomize by treatment method, a multicenter study with a larger number of cases should be conducted in the future, along with a more detailed study using multivariate analysis or propensity score matching analysis. Despite these limitations, we believe that this study is meaningful in suggesting the need to select a subsequent treatment method according to the degree of ENE and reexamine the additional benefits of cisplatin in oral cancer treatment after recurrence. Future research needs to be conducted on predictive molecular markers in different ENE subgroups, comparisons of quality of life (QOL) outcomes among different treatments, long-term follow-ups over five years to assess late complications and survival, and novel targeted therapies and immunotherapy for ENE-positive cases. These findings are expected to provide new treatment options.

## Conclusions

This retrospective study investigated the efficacy of postoperative treatment for patients with OSCC at high risk of recurrence due to ENE or positive margins. Fifty-two patients were enrolled, with 28 having ENE only, 17 with positive margins only, and seven with both. The study suggests that patients with ENEma require additional treatment with RT or CRT due to their poor prognosis, while re-resection may improve the prognosis in patients with positive margins. Future research should investigate predictive markers, QoL across treatments, long-term outcomes, and novel therapies for ENE-positive cases, aiming to provide new treatment options.
